# Allele-specific expression in a family quartet with autism reveals mono-to-biallelic switch and novel transcriptional processes of autism susceptibility genes

**DOI:** 10.1038/s41598-018-22753-4

**Published:** 2018-03-09

**Authors:** Chun-Yen Lin, Kai-Wei Chang, Chia-Yi Lin, Jia-Ying Wu, Hilary Coon, Pei-Hsin Huang, Hong-Nerng Ho, Schahram Akbarian, Susan Shur-Fen Gau, Hsien-Sung Huang

**Affiliations:** 1 Graduate Institute of Brain and Mind Sciences, College of Medicine, National Taiwan University, Taipei, 10051 Taiwan; 2 Department of Pediatrics, Yong-He Cardinal Tien Hospital, Taipei, Taiwan; 3 Department of Psychiatry, University of Utah School of Medicine, Salt Lake City, UT 84108 USA; 4 Department of Pathology, National Taiwan University Hospital and College of Medicine, National Taiwan University, Taipei, 10051 Taiwan; 5 Department of Obstetrics and Gynecology, National Taiwan University Hospital and College of Medicine, National Taiwan University, Taipei, 10051 Taiwan; 6 Graduate Institute of Medical Genomics and Proteomics, College of Medicine, National Taiwan University, Taipei, 10051 Taiwan; 7 Department of Psychiatry, Icahn School of Medicine at Mount Sinai, NY, 10029 USA; 8 Department of Psychiatry, National Taiwan University Hospital and College of Medicine, National Taiwan University, Taipei, 10051 Taiwan; 9 Neurodevelopment Club in Taiwan, Taipei, 10051 Taiwan

## Abstract

Autism spectrum disorder (ASD) is a highly prevalent neurodevelopmental disorder, and the exact causal mechanism is unknown. Dysregulated allele-specific expression (ASE) has been identified in persons with ASD; however, a comprehensive analysis of ASE has not been conducted in a family quartet with ASD. To fill this gap, we analyzed ASE using genomic DNA from parent and offspring and RNA from offspring’s postmortem prefrontal cortex (PFC); one of the two offspring had been diagnosed with ASD. DNA- and RNA-sequencing revealed distinct ASE patterns from the PFC of both offspring. However, only the PFC of the offspring with ASD exhibited a mono-to-biallelic switch for *LRP2BP* and *ZNF407*. We also identified a novel site of RNA-editing in *KMT2C* in addition to new monoallelically-expressed genes and miRNAs. Our results demonstrate the prevalence of ASE in human PFC and ASE abnormalities in the PFC of a person with ASD. Taken together, these findings may provide mechanistic insights into the pathogenesis of ASD.

## Introduction

Autism spectrum disorder (ASD) is a heritable neurodevelopmental disorder characterized by social difficulties, communication challenges, and repetitive behaviors^1^. ASD has been identified in 1 out of 68 children^2^, which reveals its high prevalence and indicates its importance as a public health issue. There is no definitive biomarker for ASD and no widely accepted treatment. Although medications can provide an improvement in behaviors for persons with ASD, they cannot reliably ameliorate all of the core symptoms of this disorder^3,4^. This limitation of therapeutic efficacy is due to the heterogeneous and multifactorial causes of ASD, which include genetic components, environmental insults, and gene-environment interactions^5,6^. Altered gene expression in the brain has been consistently identified in persons with ASD through genome-wide analysis^7–10^. In addition, phenotypic and genetic variations are entangled, which further hinders deciphering the etiology of ASD^11,12^. Although a single ultimate neuropathological feature in the brains of individuals with ASD may be impossible to define, we now know that common variant of small effect and rare *de novo* variants of large effect can combine to influence the risk for ASD. Apart from *de novo* mutations, copy number variations^13–15^, and aberrant microRNA profiles^16^, epigenetic mechanisms such as variations in DNA methylation on differential gene expression have been proposed to play a pivotal role in ASD^17–19^. Although Fragile X syndrome is the most well-known single-gene disorder, it only accounts for approximately 5% of all ASD cases^20^; the cause of most cases of ASD are unclear. Moreover, the challenge of investigating the causes is further complicated by the complex nature of ASD.

Allele-specific expression (ASE) is an unequal expression of alleles. One extreme example is mono-allelic expression (MAE), in which one allele is expressed, while the other is inactive. MAE consists of several different mechanisms, such as genomic imprinting^21^, X chromosome-inactivation^22^, expression quantitative trait loci (eQTL)^23,24^, and autosomal random MAE^25^. Genomic imprinting is essential for neurodevelopment and viability of the organism^26^. Mono-allelically expressed genes play important roles during development, and they are predisposed to loss of their function through mutations, thus contributing to diseases^27,28^. Indeed, random or stochastic monoallelically expressed genes are enriched for candidate genes for neurodevelopmental disorders^29^. Furthermore, MAE affects epigenetic processes in brains of individuals with ASD^30^ and dysregulated genomic imprinting has been identified in persons with ASD^31,32^.

The quality of genetic information can be improved by analyzing complete genome sequences from family members, which was demonstrated by the pioneering work of Roach *et al*. (2010) using a family quartet (two siblings and their parents)^33^. Moreover, information regarding single nucleotide polymorphisms (SNPs) from the parent is critical for parent-of-origin allele-specific expression analysis, although haplotype phasing in an outbred human population is complicated^34^. Therefore, it is necessary to acquire samples from a family quartet to determine the parental source of transcripts in offspring. Despite the relevance of MAE to ASD, comprehensive analysis of MAE in a family quartet with ASD has not been conducted due to limited access to samples.

To address this critical knowledge gap, we investigated the role of ASE in the pathogenesis of ASD using special human parent-child quartet samples. Genomic DNA from parent and offspring and RNA from offspring’s postmortem prefrontal cortex (PFC) of the brain were analyzed. One offspring had been diagnosed with ASD. We observed distinct ASE patterns of genes and microRNAs (miRNAs) in the PFC of both offspring. Importantly, we found a mono-to-biallelic switch for *LRP2BP* (LRP2 binding protein) and *ZNF407* (Zinc finger protein 407) in the offspring diagnosed with ASD. We also found a novel site of RNA editing in *KMT2C* (Lysine (K) methyltransferase 2 C); a novel development stage- and brain-specific maternally-expressed gene, *DUSP22* (Dual specificity phosphatase 22); and a novel development stage-specific paternally-expressed miRNA, *miR-335*, in the PFC of both offspring. *KMT2C*, *DUSP22* and *miR-335* are autism susceptibility genes and miRNAs. Our results provide further evidence that ASE could contribute to ASD.

## Results

### Quality and quantity of DNA and RNA of a parent-child quartet with ASD met the requirements for deep sequencing

**Figure 1:**
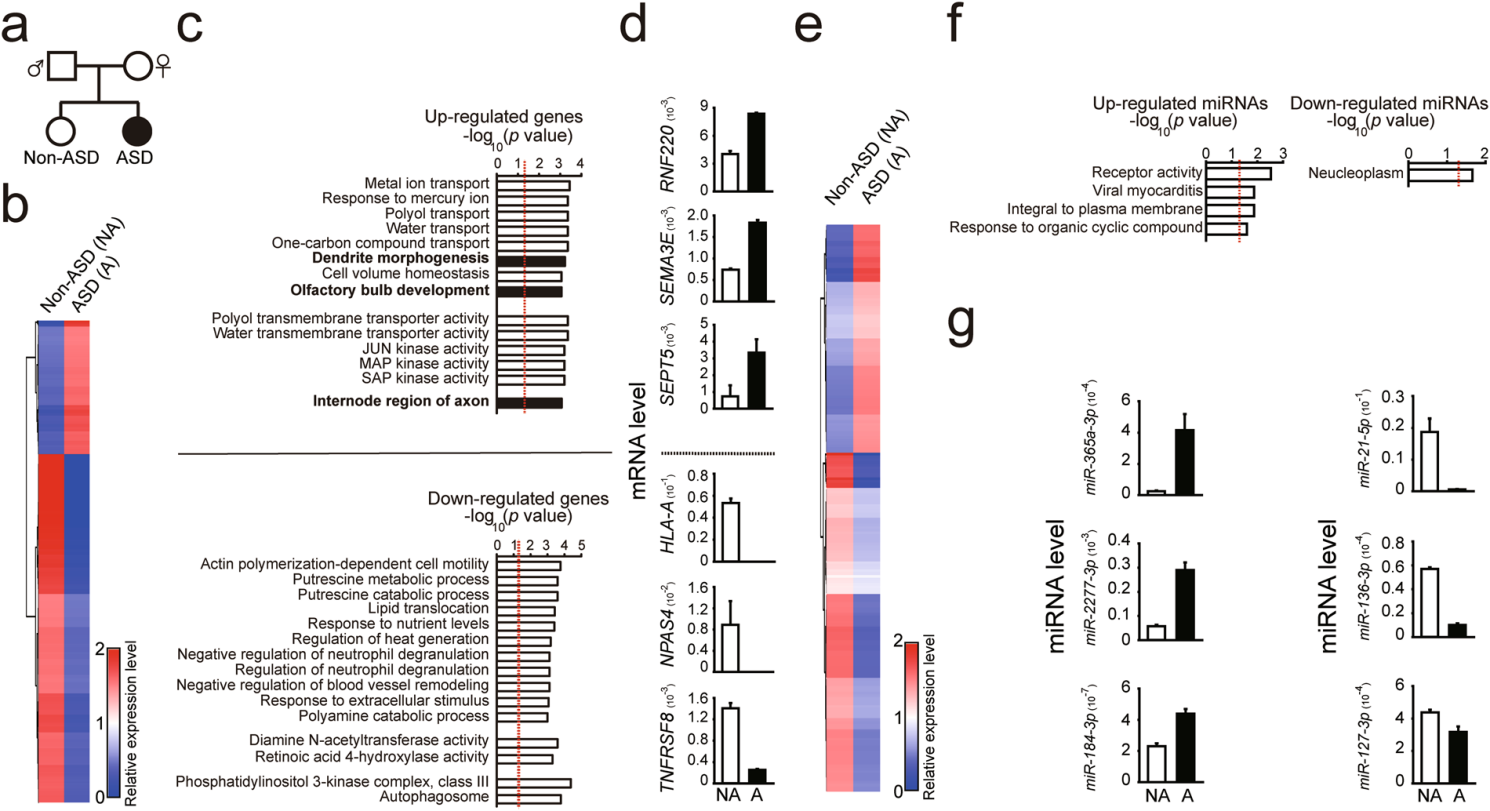
Differential gene and miRNA expression patterns in the postmortem prefrontal cortex (PFC) of a person without and with ASD. (**a**) Schematic diagram of the family tree from a family quartet with ASD. Square indicates male and circle indicates female. Black solid circle indicates the offspring with ASD; open circle indicates the offspring without ASD. (**b**) Heatmap analyses showing gene expression profiles for the offspring without and with ASD. (**c**) Gene ontology (GO) enrichment analysis was performed for affected genes from the offspring with ASD. (**d**) The top genes that were up-regulated (top) and down-regulated (bottom) were validated by qPCR; expression levels of genes for the offspring with ASD (black bars) compared with levels for the offspring without ASD (open bars). (**e**) Heatmap analyses showing miRNA expression profiles for the offspring without and with ASD. (**f**) Gene ontology (GO) enrichment analysis was performed for affected miRNAs from the offspring with ASD. (**g**) The top miRNAs that were up-regulated (top) and down-regulated (bottom) were validated by qPCR; expression levels of miRNA in the offspring with ASD (black bars) compared with levels in the offspring without ASD (open bars). NA, the offspring without ASD; A, the offspring with ASD. The data points above the red dashed line represent *P* values less than 0.05. Euclidean distance was used to generate the heatmap plots.

We analyzed ASE on a genome-wide scale and investigated whether dysregulated ASE occurs in persons with ASD using samples from a family quartet with ASD consisting of genomic DNA from the parent and offspring and RNA extracted from postmortem PFC of the offspring (Fig. 1a). Sequencing of parental genomic DNA provided information regarding single nucleotide polymorphisms (SNPs), which is essential for determining the parental source of offspring’s transcripts. In this family quartet, both offspring were female, and one had been diagnosed with ASD (Fig. 1a). Supplementary Fig. 1 provides a more detailed pedigree of this family, which shows epilepsy and deafness co-occurred in both the affected and unaffected offspring in addition to other familial health conditions. Next, we performed DNA sequencing of parents and offspring; RNA-sequencing and follow-up transcriptomic and ASE analysis was performed on the offspring. The detailed demographic and deep sequencing information is presented in Supplementary Table 1. The quantity and quality of genomic DNA from parents and offspring met the requirements for DNA-sequencing (Fig. S2a); in addition, the quantity and quality of the offspring’s RNA met the requirements for RNA-sequencing (Fig. S2b). Total read number and mappability of the offspring’s RNA-Seq data met the requirements for further statistical analysis (Supplementary Table 1). Taken together, the DNA and RNA from the offspring qualified for deep sequencing.

### Gene and miRNA expression are altered in the PFC of the offspring with ASD

To determine whether dysregulated gene expression was present in the postmortem PFC of offspring with ASD, we compared gene expression patterns of the offspring with and without ASD on a genome-wide scale. We focused on genes with expression levels of more than 0.3 FPKM in the offspring without ASD and a fold change between the offspring with and without ASD larger than 0.5 or less than −0.5, on a base-10 logarithmic scale (Fig. 1b and Supplementary Table 2). Rather than use the typical two-fold change, we used a fold change of 3.16 (=10^(0.5)), which is a more stringent threshold. When compared to the offspring without ASD, we detected 2293 up-regulated and 5980 down-regulated gene isoforms in the PFC of the offspring with ASD. We then performed gene ontology (GO) enrichment analysis on the affected gene isoforms to further investigate which functions, processes, and components were affected. Focusing on gene isoforms involved in brain-related functions, we observed that genes related to the development of dendrites, axons and the olfactory bulb were affected (Fig. 1c and Supplementary Table 3). None of the GO terms survived after false discovery rate (FDR) correction. The functions of the genes related to olfactory bulb development (*ID2*, *AGTPBP1* and *SEMA3A*) are related to overall brain development^35–37^. If we applied more stringent criteria (log_2_ (ASD/non-ASD > 2 or −2 and q value < 0.05), we only detected 67 genes with altered expression in the offspring with ASD (Supplementary Table 4). Due to the small size of the genes, GO analysis could not be further pursued. To validate the accuracy of our heatmap results, we confirmed the expression levels of the top genes that were up-regulated (such as *RNF220*, *SEMA3E*, and *SEPT5*) and down-regulated (such as *HLA-A*, *NPAS4*, and *TNFRSF8*) with qPCR (Fig. 1d). These genes were chosen based on the availability of reliable primers, high expression levels, and a difference in expression level between ASD and non-ASD samples. To examine if the altered genes could be observed in other ASD cohorts, we compared our altered genes with RNA-Seq data from Dr. Weinberger’s group^7^ for the dorsolateral prefrontal cortex from three persons with ASD and three matched controls. We observed that expression of 2406 out of 8273 genes was also altered in this ASD cohort (Fig. S3a and Supplementary Table 5). For analysis of Dr. Weinberger’s RNA-Seq data, we used the same criteria of altered gene expression as we used for our ASD cohort. To investigate if the altered genes could be observed in a larger ASD cohort, we compared our altered genes with RNA-Seq data from a study by Dr. Geschwind’s group^10^ of the cortex from persons with ASD and matched controls. We found that expression of 387 out of 1087 genes was also altered in this ASD cohort (Supplementary Table 6).

Small RNAs, such as miRNAs, affect gene expression, but whether dysregulated miRNAs contribute to the etiology of ASD has not been well studied. To address this question, we compared the expression levels of miRNAs from the PFC of the two offspring on a genome-wide scale. We focused on miRNAs with a fold change between the offspring with and without ASD larger than 0.5 or less than −0.5, on a base-10 logarithmic scale, which resulted in a threshold of fold change of 3.16 (=10^(0.5)). When PFC miRNAs from the offspring with ASD were compared with those of the offspring without ASD, we found 105 up-regulated and 125 down-regulated miRNAs in the offspring diagnosed with ASD (Fig. 1e and Supplementary Table 7). We then performed GO analysis on the affected miRNAs (Fig. 1f and Supplementary Table 8) and examined genes targeted by altered miRNAs (Supplementary Table 9). None of GO terms survived after FDR correction. To validate the accuracy of our heatmap results, we performed qPCR to confirm the expression levels of the top up-regulated miRNAs (such as *miR365a-3p*, *miR-2277-3p*, and *miR-184-3p*) and down-regulated miRNAs (such as *miR-21-5p*, *miR-136-3p*, and *miR-127-3p*) (Fig. 1g). These miRNAs were chosen based on the availability of reliable primers, high expression levels, and a difference in expression level between ASD and non-ASD samples. To determine whether those altered miRNAs could be detected in other ASD cohorts, we compared our altered miRNAs with RNA-Seq data from Dr. Weinberger’s group^7^ for the dorsolateral prefrontal cortex of three persons with ASD and the three matched controls, using same criteria for analysis as we used in our ASD cohort. We observed that expression of 12 out of 210 miRNAs was also altered in this ASD cohort (Fig. S3b and Supplementary Table 5). To investigate if the altered miRNAs could be detected in a larger ASD cohort, we compared our altered miRNAs with RNA-Seq data from a study by Dr. Geschwind’s group^16^ of the cortex from persons with ASD and matched controls. We found that expression of 1 out of 58 miRNAs was also altered in this ASD cohort (shaded genes, Supplementary Table 6). Taken together, our results showed the PFC of the offspring with ASD contained genes and miRNAs with altered expression.

### Autism susceptibility genes were altered in the PFC of the offspring with ASD

**Figure 2:**
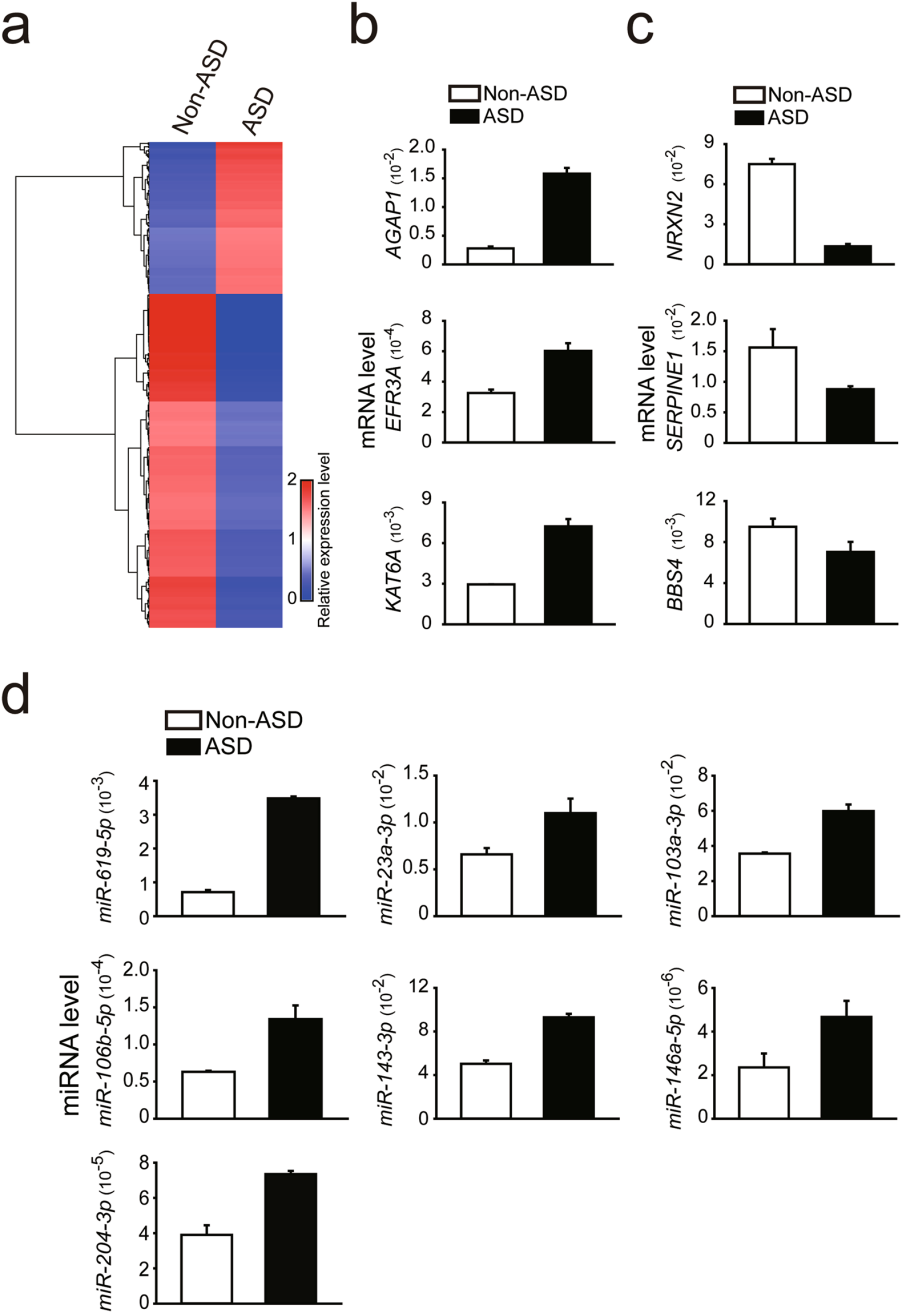
Distinct expression patterns of autism susceptibility genes and miRNAs from postmortem prefrontal cortex (PFC) of the offspring diagnosed with ASD. (**a**) Heatmap analyses showing different gene expression profiles of autism susceptibility genes from the PFC of the offspring without and with ASD. The top genes that were up-regulated (**b**) and down-regulated (**c**) were validated by qPCR; expression levels in the offspring with ASD (black bars) compared with levels in the offspring without ASD (open bars). (**d**) Autism susceptibility miRNAs were determined with qPCR for the offspring without ASD (open bars) and the offspring with ASD (black bars). NA, the offspring without ASD; A, the offspring with ASD. Euclidean distance was used to generate the heatmap plots.

To investigate whether autism susceptibility genes were preferentially altered, we compared expression levels of autism susceptibility genes in the postmortem PFC of the two offspring. The source of autism susceptibility genes was from the Simons Foundation Autism Research Initiative (SFARI, September 2016; Supplementary Table 10). We focused on genes with expression levels of more than 0.3 FPKM in the offspring without ASD and a fold change between the offspring with and without ASD larger than 0.5 or less than −0.5, on a base-10 logarithmic scale and as a result, the threshold for fold change used was 3.16 (=10^(0.5)). Compared with the offspring without ASD, the PFC of the offspring with ASD exhibited 142 up-regulated and 312 down-regulated autism susceptibility gene isoforms (Fig. 2a and Supplementary Table 11). We performed a hypergeometric test to assess whether the above overlaps were more than one would expect by chance and found significant overlaps (*p* < 0.001). To validate the heatmap results regarding differences in regulation of these gene isoforms in the offspring with ASD, we compared the offspring’s top up-regulated autism susceptibility genes (such as *AGAP1*, *EFR3A* and *KAT6A*, Fig. 2b) and down-regulated autism susceptibility genes (such as *NRXN2*, *SERPINE1*, and *BBS4*, Fig. 2c). These genes were chosen based on the availability of reliable primers, high expression levels, and a difference in expression level between ASD and non-ASD samples. To determine whether the altered ASD susceptibility genes could be observed in other ASD cohorts, we compared our altered ASD susceptibility genes with RNA-Seq data from Dr. Weinberger’s group^7^ for the dorsolateral prefrontal cortex of three persons with ASD and three matched controls. We observed altered expression in 51 out of 455 genes in this ASD cohort (Fig. S3c and Supplementary Table 5). For analysis of the data in the cohort from Dr. Weinberger’s data, we used the same criteria for the ASD susceptibility genes we used in our ASD cohort. To investigate if the altered genes could be observed in a larger ASD cohort, we compared our altered genes with RNA-Seq data from a study by Dr. Geschwind’s group^10^ of the cortex from persons with ASD and matched controls. We found that expression of 29 out of 1087 genes was also altered in this ASD cohort (shaded genes, Supplementary Table 6).

When compared with the offspring without ASD, the following autism susceptibility miRNAs^16,38^ were altered in the offspring with ASD: *miR-619-5p*, *miR-23a-3p, miR-103a-3p, miR-106b-5p, miR-143a-3p, miR-146a-5p*, and *miR-204-3p* (Fig. 2d and Supplementary Table 12). A hypergeometric test showed the above overlaps were not significant (*p* = 0.92). Since miRNAs regulate gene expression, we also examined genes targeted by the affected miRNAs (Supplementary Table 12). We then performed gene ontology (GO) enrichment analysis on the affected miRNA target genes to further investigate which functions and pathways were affected. However, we did not observe any significant results. Our data showed altered expression of autism susceptibility genes from SFARI in the PFC from the offspring with ASD.

### Allele-specific gene expression was altered in the postmortem PFC of the offspring with ASD

**Figure 3:**
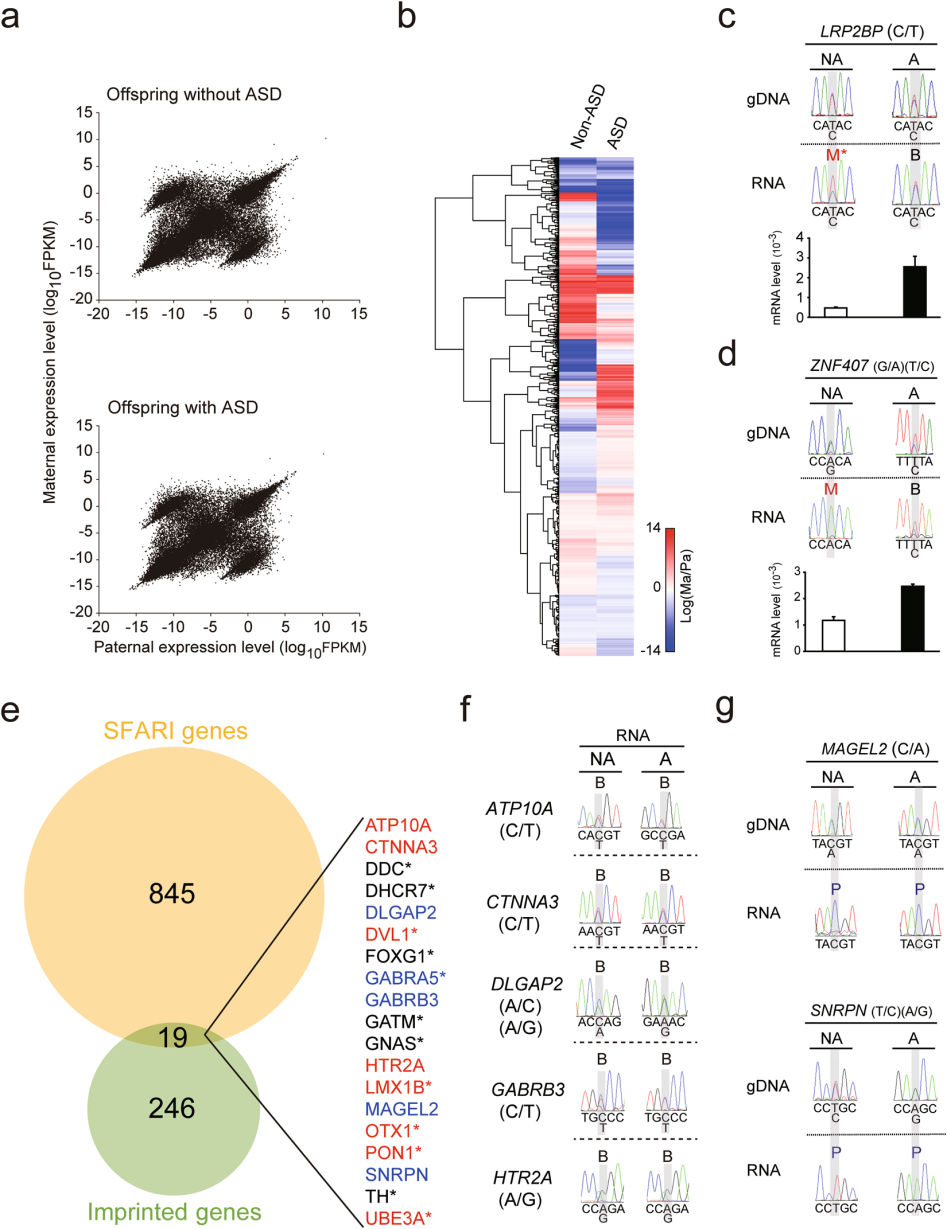
Distinct patterns of allele-specific gene expression in postmortem prefrontal cortex (PFC) of the offspring without and with ASD. (**a**) Parental expression patterns from the PFC of the offspring without ASD (top) and with ASD (bottom) analyzed on a genome-wide scale with RNA-Seq. (**b**) Heatmap analyses showing allele-specific gene expression profiles in the offspring without and with ASD. (**c**) Sanger sequencing validated the allele-specific expression of *LRP2BP* from the two offspring. Expression levels of *LRP2BP* from the PFC of the offspring without ASD (open bar) and with ASD (black bar) were quantified by qPCR. (**d**) Sanger sequencing validated the allele-specific expression of *ZNF407* from the two offspring. Expression levels of *ZNF407* from the PFC of the offspring without ASD (open bar) and with ASD (black bar) were quantified by qPCR. The offspring with and without ASD each had unique SNPs for *ZNF407*: G/A for the offspring without ASD and T/A for the offspring with ASD. (**e**) Venn diagram showing overlapped Simons Foundation Autism Research Initiative (SFARI) genes with known human imprinted genes. (**f**,**g**) The imprinting status of seven of the 19 overlapped genes was verified by Sanger sequencing. NA, the offspring without ASD; A, the offspring with ASD; B, bi-allelic; M, maternal; p, paternal. *Gene could not be validated due to lack of availability of SNPs or low gene expression levels. SNP information was shown as (paternal allele/maternal allele). Euclidean distance was used to generate the heatmap plots.

To examine allele-specific gene expression and to determine whether dysregulated allele-specific gene expression occurred in the offspring with ASD, the parents’ and offspring’s genomic DNA was analyzed with DNA-Seq, and the offspring’s postmortem PFC RNA was analyzed with RNA-Seq followed by ASE analysis. First, we observed a distinct allele-specific gene expression pattern, which contained a diagonal line with one paternally-dominant cohort and one maternally-dominant cohort in both offspring (Fig. 3a and Supplementary Table 13). Genes within the diagonal line indicate both of their alleles were expressed equally, which represented the majority of genes; genes above or below the diagonal line indicate genes that were expressed predominantly from either maternal or paternal allele. This pattern has been observed consistently in different brain regions related to the mouse visual system^39^ as well as in cell types of the mouse visual cortex^40^. In contrast, when we compared the ASE patterns of all of SFARI’s autism susceptibility genes, several genes differed between the offspring with and without ASD (Fig. 3b and Supplementary Table 14). A hypergeometric test demonstrated these differences in genes between siblings were not by chance (*p* < 0.001). We validated allele-specific expression for our candidate genes with Sanger sequencing and determined *LRP2BP* and *ZNF407* were both mono-allelically expressed in the offspring without ASD, but bi-allelically expressed in the offspring with ASD (Fig. 3c,d, top). *LRP2BP* was also monoallelically expressed in other non-ASD brain samples (Fig. S4a). This mono-to-biallelic switch reflects their expression levels (Fig. 3c,d, bottom). Because there is an overlap between autism susceptibility genes from SFARI (Supplementary Table 10, September 2016) and known human imprinted genes from the Geneimprint website (Supplementary Table 15, September 2016) (Fig. 3e), we validated seven of the 19 overlapped genes with Sanger sequencing (Fig. 3f,g). Due to the lack of availability of SNPs and low gene expression levels, the remaining overlapped genes could not be validated. For genes that could be validated, Sanger sequencing showed *ATP10A, CTNNA3, DLGAP2, GABRB3*, and *HTR2A* were not imprinted in either of the offspring (Fig. 3f), whereas *MAGEL2* and *SNRPN* were imprinted in both (Fig. 3g). The imprinting status of *ATP10A*, *DLGAP2*, and *HTR2A* was further confirmed in other brain samples (Fig. S4b). Taken together, our data suggest that allele-specific gene expression occurs in human PFC and dysregulated allele-specific gene expression occurred in the PFC of the offspring with ASD.

### Allele-specific miRNA expression was altered in the PFC of the offspring with ASD

**Figure 4:**
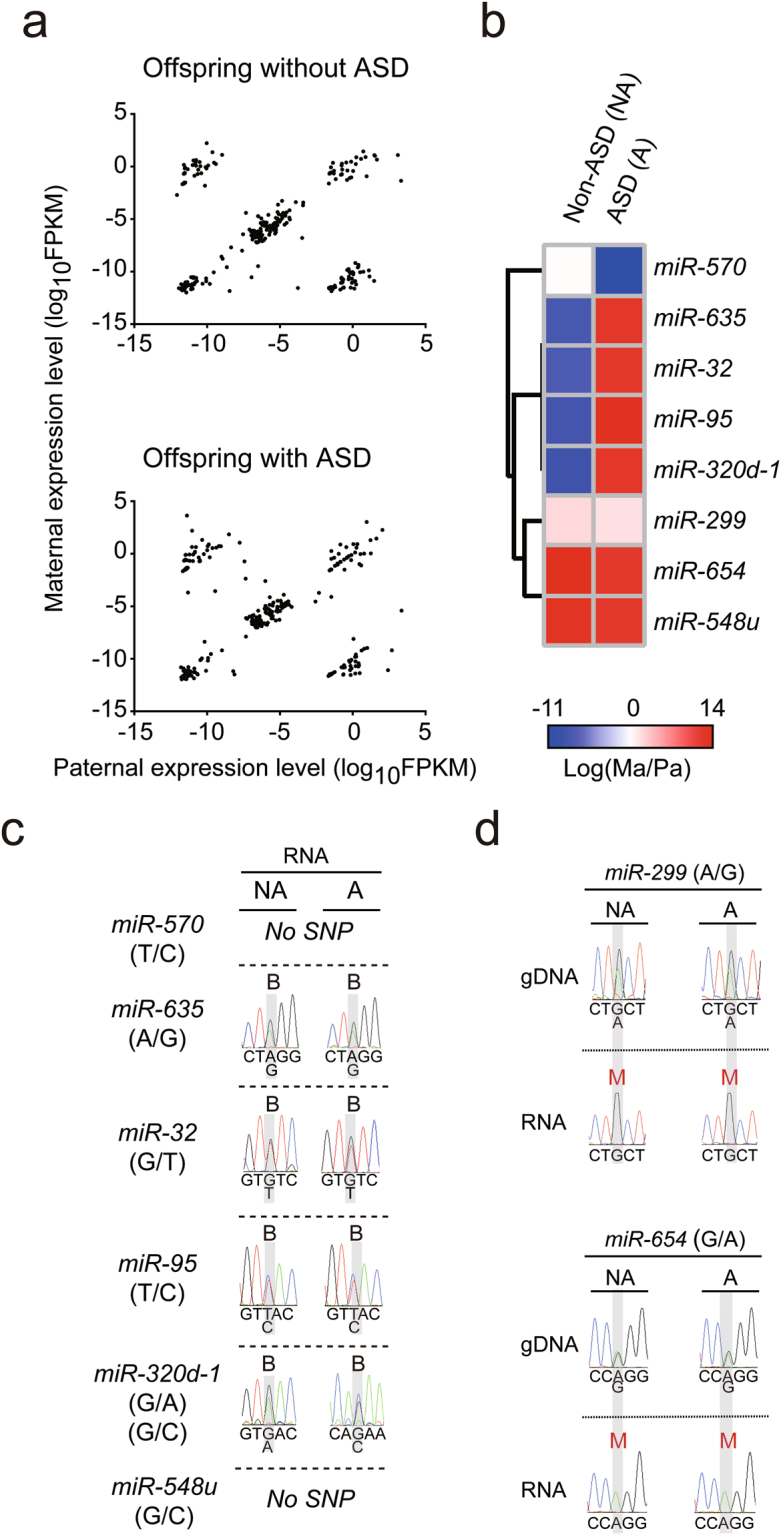
Distinct patterns of allele-specific miRNAs in the postmortem prefrontal cortex (PFC) of offspring without and with ASD. (**a**) Parental expression pattern of miRNAs was analyzed on a genome-wide scale with RNA-Seq in the PFC of both offspring. (**b**) Heatmap analyses of allele-specific expression profiles for miRNAs with fold changes of >0.5 or <−0.5 on a base-10 logarithmic scale between the offspring without and with ASD. (**c**,**d**) Allele-specific expression was validated for the miRNAs in (**b**) from the two offspring by Sanger sequencing. SNP information is shown as (paternal allele/maternal allele). Euclidean distance was used to generate the heatmap plots.

To determine whether allele-specific expression of miRNAs occurs in the PFC of humans and whether the expression is dysregulated in persons with ASD, we profiled allele-specific miRNA expression in the PFC of the offspring with and without ASD. We observed a pattern of ASE for miRNAs, which showed a diagonal line with a paternally-dominant cohort and a maternally-dominantly cohort (Fig. 4a), similar to that seen for genes (Fig. 3a). In addition, this pattern has been observed consistently in different brain regions related to the mouse visual system^39^. Heatmap clustering compared miRNAs from the two offspring whose fold change on a base-10 logarithmic scale was larger than 0.5 or less than −0.5 (Fig. 4b and Supplementary Table 16), and as a result, the threshold for fold change used was the 3.16 (=10^(0.5)). We validated the allele-specific miRNA expression with Sanger sequencing (Fig. 4c,d). There was no difference in allele-specific miRNA expression between offspring without and with ASD. However, we identified maternally-expressed miRNAs (*miR-299* and *miR-654*) in both offspring (Fig. 4d). In summary, our data show distinct patterns of allele-specific miRNA expression in the PFC of both offspring.

### Genomic map of parent-of-origin-specific gene and miRNA expression in the human PFC

**Figure 5:**
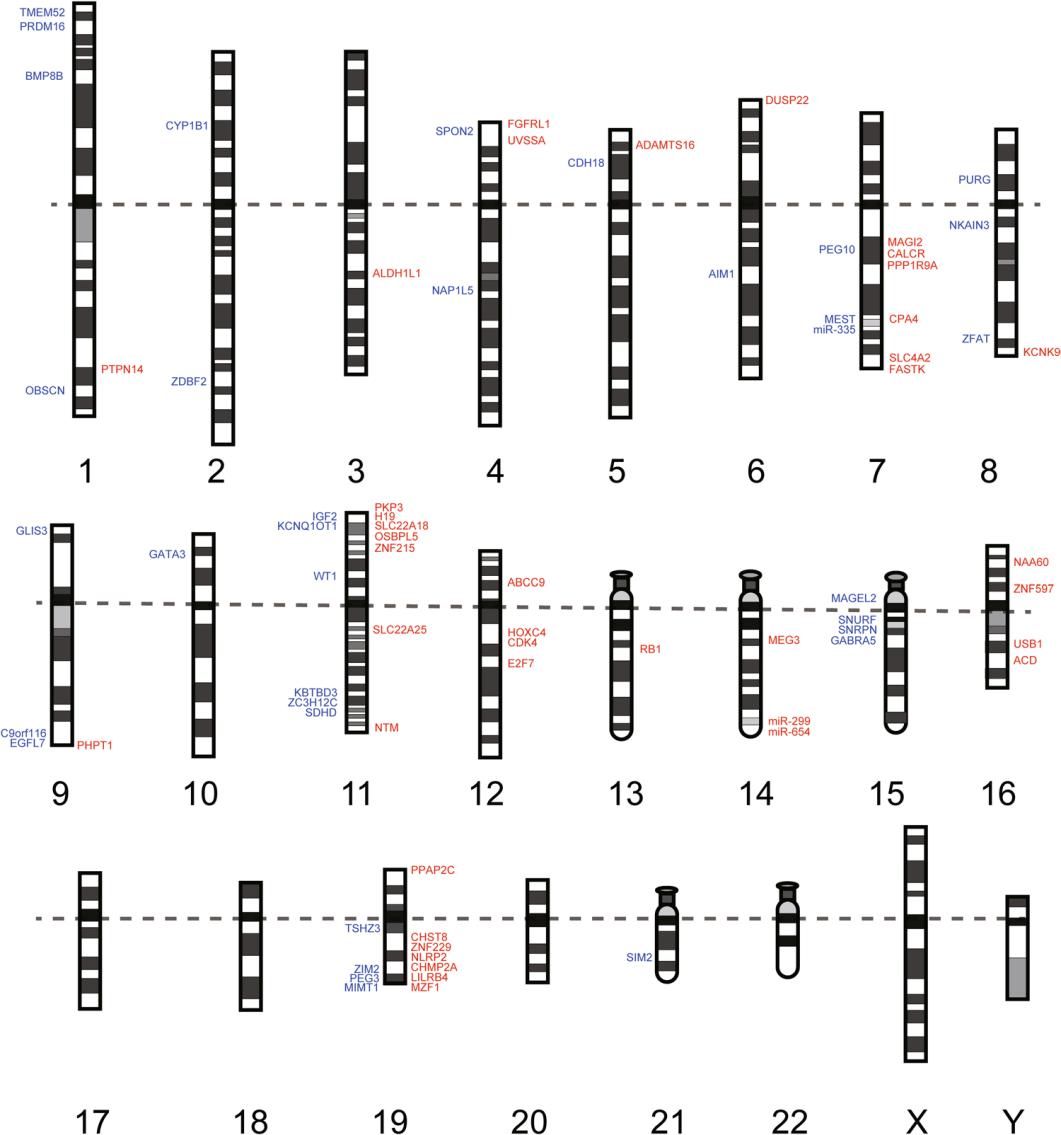
Chromosome map of parent-of-origin-specific genes and miRNAs in the PFC of the offspring without ASD. Examination of 23 chromosomes for the presence of paternally- (blue) and maternally- (red) expressed imprinted genes and miRNAs in the PFC from the offspring without ASD. Dotted line separates the p and q arm of each chromosome.

**Figure 6:**
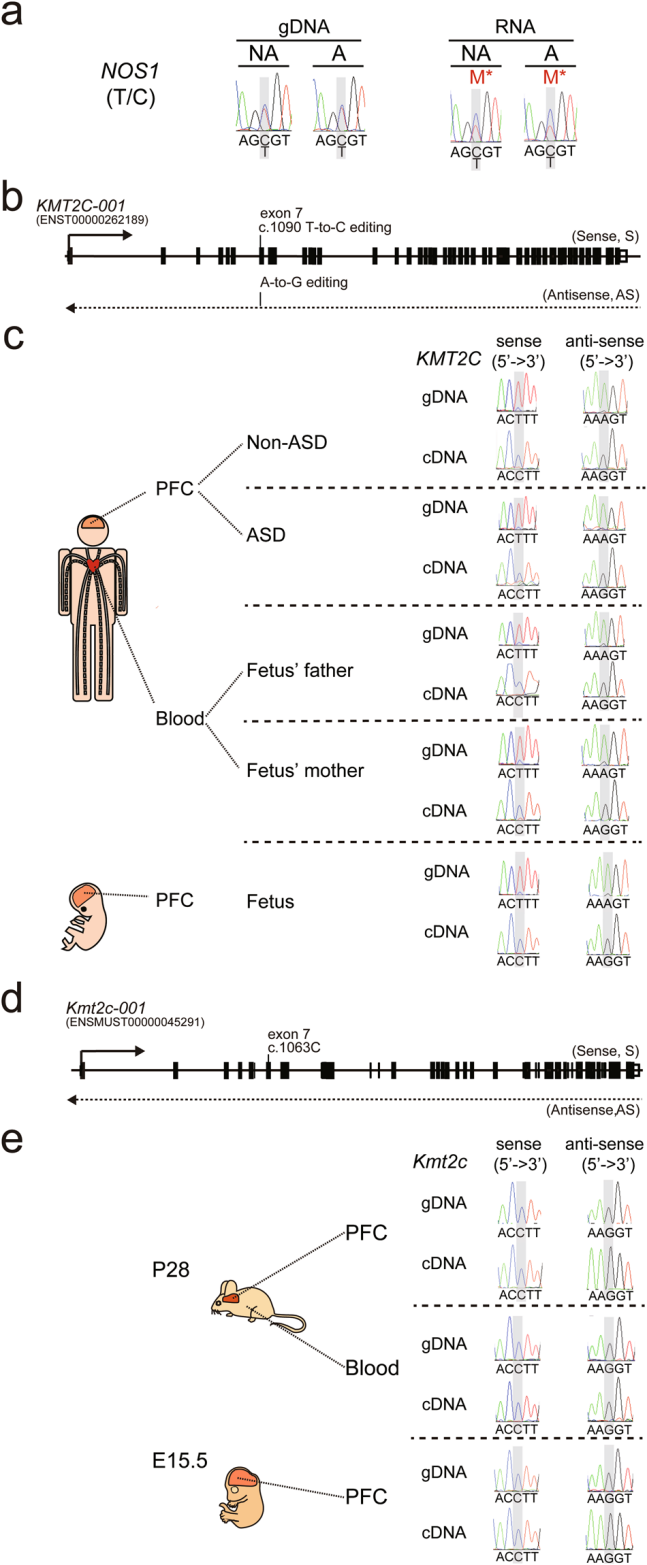
Identification of noncanonical imprinting in the PFC of the offspring without and with ASD and editing of *KMT2C* transcript in human PFC. (**a**) Sanger sequencing was used to analyze noncanonical imprinting of *NOS1* in the PFC of the offspring without and with ASD. “M*” stands for maternally-biased expression. (**b**) Schematic diagram of the genomic locus of human *KMT2C*. Arrows indicate the direction of transcription. (**c**) Sanger sequencing was performed to analyze RNA editing for *KMT2C* in the PFC of the offspring without and with ASD, fetal PFC, and blood from parents of the fetus. (**d**) Schematic diagram of the genomic locus of mouse *Kmt2c*. Arrows indicate the direction of transcription. (**e**) Sanger sequencing was performed to analyze RNA editing for *Kmt2c* in PFC and blood from postnatal day 28 (P28) mice and PFC from embryonic day 15.5 (E15.5) mice. SNP information is shown as (paternal allele/maternal allele).

To map parent-of-origin-specific gene and miRNA expression on a genome-wide scale in human PFC, we compared our data obtained from the PFC of the offspring without ASD for allele-specific gene and miRNA expression with known human imprinted genes reported by the Geneimprint website (Supplementary Table 15, September 2016) and previous literature^41^. RNA-Seq and follow-up ASE analysis confirmed the presence of 60% of known paternally expressed genes and 60% of known maternally expressed genes (Supplementary Fig. 5 and Table 17). We mapped the confirmed imprinted genes and miRNAs into 23 human chromosomes (Fig. 5). This mapping identifies the parent-of-origin-specific genes and miRNAs expressed on a genome-wide scale in the human PFC. Canonical genomic imprinting involves silencing of the maternal and paternal allele. In contrast, noncanonical genomic imprinting involves maternal or paternal allele expression biases^42^. We identified a noncanonical imprinted gene, *NOS1* (Fig. 6a) in the PFC of both offspring.

### Novel transcriptional processes of autism susceptibility genes were identified in human PFC

**Figure 7:**
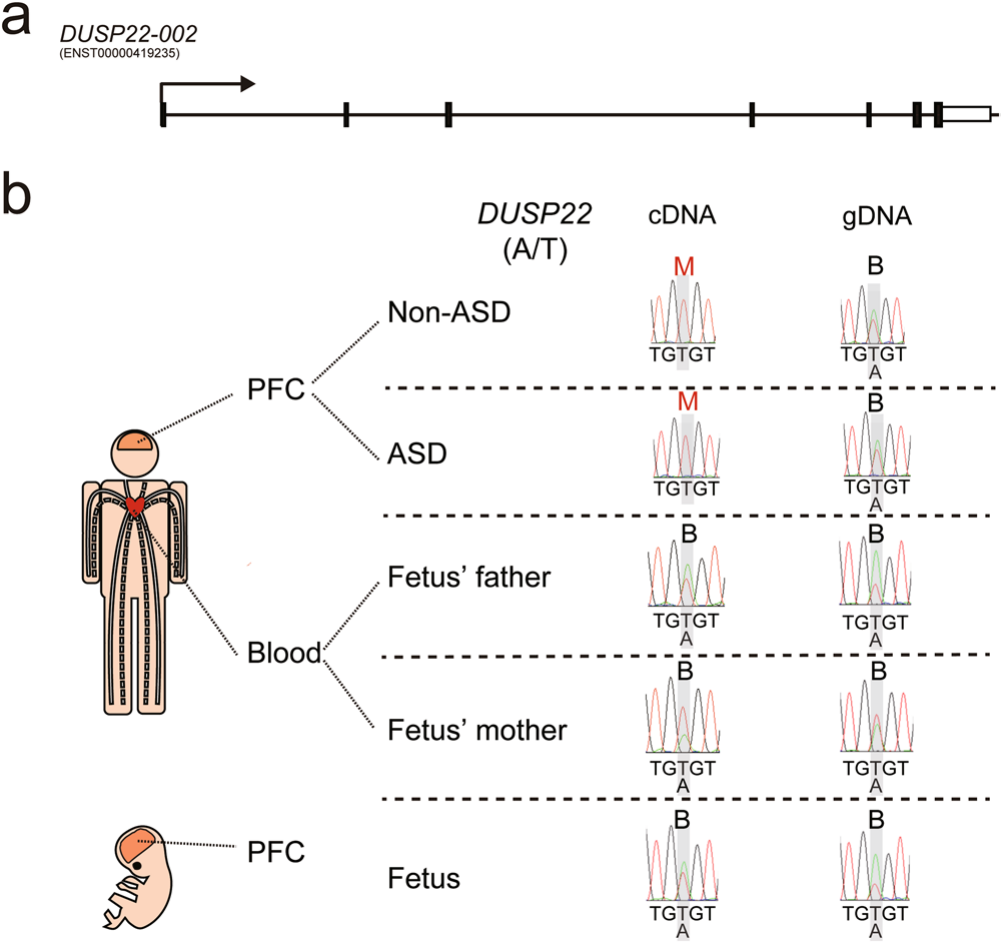
*DUSP22* is maternally expressed in the PFC of the offspring without and with ASD. (**a**) Schematic diagram of the genomic locus of *DUSP22*. Arrows indicate the direction of transcription. (**b**) Sanger sequencing was performed to analyze allele-specific *DUSP22* expression in the PFC of the offspring without and with ASD, fetal PFC, and blood from the parents of the fetus. “M” stands for maternal expression. “B” stands for biallelic expression. SNP information is shown as (paternal allele/maternal allele).

**Figure 8:**
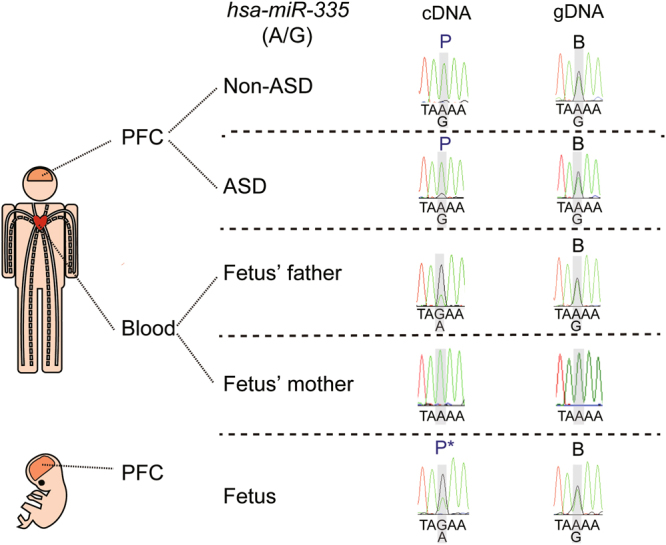
*miR-335* is paternally expressed in the PFC of the offspring without and with ASD. Sanger sequencing was performed to analyze allele-specific *miR-335* expression in the PFC of the offspring without and with ASD, fetal PFC, and blood from the parents of the fetus. “P” stands for paternal expression. “B” stands for biallelic expression. “P*” stands for paternally-biased expression. SNP information is shown as (paternal allele/maternal allele).

Validation of allele-specific expression in the PFC of both offspring with Sanger sequencing identified three novel transcriptional processes of autism susceptibility genes. First, we identified a novel human-specific site of RNA editing in *KMT2C* (Fig. 6b). When we analyzed RNA editing in human adult and fetal PFC (Fig. 6c), we observed T-to-C RNA editing of *KMT2C* sense transcript, which was in contrast to A-to-G RNA editing for *KMT2C* antisense, which could cause a phenylalanine-to-leucine change at the protein level (p.F291L). This pattern of RNA editing was not seen when we analyzed PFC from postnatal day 28 (P28) and embryonic day 15.5 (E15.5) mouse (Fig. 6d,e, respectively), suggesting this RNA editing in *KMT2C* is human-specific. These differences in RNA editing patterns between human and mouse tissue were also seen when we examined human and mouse blood (Fig. 6c,e). Second, we identified a development stage- and brain-specific maternally-expressed gene, *DUSP22* (Fig. 7). We found that *DUSP22* was maternally expressed in adult PFC but bi-allelically expressed in fetal PFC and adult blood (Fig. 7b). The imprinting status of *DUSP22* has been validated in other fetal tissue (Fig. S4b). Because we have been unable to identify the exonic SNPs in mouse *Dusp22*, we have not examined the ASE pattern of *Dusp22* in mouse PFC and blood. Finally, we identified a development stage-specific paternally-expressed miRNA, *miR-335* (Fig. 8), which was paternally-expressed in the adult PFC but paternally-biased in the fetal PFC. Taken together, our results add new information about the dynamic transcriptomic processes in the brain.

## Discussion

Our genome-wide analysis provides new information regarding expression of allele-specific genes and miRNAs in human PFC and persons with ASD. Analysis of the PFC revealed a distinct allele-specific expression pattern, which contained a diagonal line with a maternally dominant cohort and a paternally dominant cohort. Moreover, we identified novel allele-specific genes such as *DUSP22* and miRNAs such as *miR-335* in both the offspring with and without ASD, as well as a mono-to-biallelic switch for *LRP2BP* and *ZNF407* in the offspring diagnosed with ASD. We also identified a novel human-specific site of RNA editing in *KMT2C*. Importantly, our study results indicate that a genome-wide ASE map could provide a powerful model for understanding neuropsychiatric disorders through the study of key features of dynamic allele-specific gene and miRNA expression in human PFC, supplemented with the study of the roles of dysregulated allele-specific genes and miRNAs in ASD.

Dual specificity phosphatase 22 (DUSP22) is an enzyme, which activates the JNK signaling pathway^43^. JNK activation has been shown to play an essential role in organogenesis during mouse development by regulating cell survival, apoptosis, and proliferation^44^. The physiological role of *DUSP22* in the brain is unclear, and a rare *DUSP22* deletion was found in a patient with autism and mild intellectual disability^45^. *DUSP22* in the PFC from the offspring without and with ASD consistently showed maternal expression. Our finding further extends a previous finding showing *DUSP22* is a monoallelically-expressed gene^46^. Interestingly, *DUSP22* from fetal PFC showed bi-allelical expression, therefore it would be of interest to investigate how a biallelic-to-monoallelic switch is regulated for *DUSP22* during prefrontal development. Moreover, *LRP2BP* and *ZNF407* in the PFC from the offspring with ASD consistently showed a mono-to-biallelic switch. Importantly, dysregulation of *LRP2BP*^47^ and *ZNF407*^48^ has been identified in persons with ASD. However, the roles of LRP2BP and ZNF407 in the brain have not been identified and the function of LRP2BP protein is still unknown. In contrast, the function of ZNF407 protein has been shown to regulate glucose homeostasis^49^. Therefore, because glucose homeostasis is critical for normal brain function, dysregulation of ZNF407 could affect brain development and function. It would be of interest not only to investigate how a mono-to-biallelic switch is regulated for *LRP2BP* and *ZNF407*, which could provide insight into their roles in ASD specifically, but also to study the roles of LRP2BP and ZNF407 more generally during neurodevelopment. The PFC from the offspring without and with ASD consistently showed paternal expression of *miR-335*, which further extends a previous finding showing mouse *miR-335* is paternally-expressed^50^.

Lysine methyltransferase 2 C (KMT2C) has histone methylation activity and is a transcriptional coactivator. In our studies, we found that T-to-C RNA editing occurred in the *KMT2C* sense transcript. This editing causes a missense mutation, which converts phenylalanine to leucine at amino acid 291 of *KMT2C*. Phenylalanine and leucine are both neutral and non-polar amino acids, therefore, this change should have a minor effect on the protein structure of KMT2C. Indeed, when we used SIFT (http://sift.jcvi.org/) to predict whether such amino acid substitution affects protein function, the result predicted that this change is a tolerated substitution. It would be of interest to investigate the physiological significance of the T-to-C editing event in the sense transcript and an A-to-G editing event in the antisense transcript.

It has been reported that parent-of-origin-specific expression is also brain-region specific^51^. Our results demonstrate expression of parent-of-origin-specific genes and miRNAs in human PFC, which has not previously been reported. Our transcriptomic and ASE analysis of the PFC of the offspring with and without ASD of a family quartet identified dysregulated gene and miRNA expression, and ASE in the offspring with ASD, which could identify a novel set of autism susceptibility genes and miRNAs. It will be important to identify the regulatory mechanisms for dysregulated transcriptomic and allele-specific expression of these genes and miRNAs identified in the offspring with ASD. To this end, it will be essential to determine if stringent identification of allele-specific genes together with the systematic screening of allele-specific chromatin and DNA modifications could unravel markers for further mechanistic validation. It would also be interesting to further investigate whether cis-transcribed non-coding RNAs, intra-nuclear allelic positioning, and chromosomal interactions are associated with the allele-specific expression.

Several methods are available for seeking the allele-specific expressed genetic locus on a genome-wide scale, which include *in silico* prediction pipelines^52^, SNP genotyping arrays^53^, gene expression arrays^54^ and transcriptome sequencing approach^55^. The transcriptomic approach is based on detecting allelic expression with RNA-Seq reads that map heterozygous SNPs, where the identity of the base is used to distinguish allelic origin and a reciprocal cross is used to discriminate parent-of-origin specificity from strain-specific or random biases. The transcriptomic approach for allele-specific gene expression is a paradigm shift in comparison to previous methods. However, recent literature has indicated a high FDR could explain the majority of novel imprinted genes in an RNA-Seq approach resulting from the contribution of several factors. First, systematic errors in technical and biological replicates include priming, fragmentation, and PCR biases that arise during library construction and sequencing. One can adopt a mock reciprocal cross as a negative control to set a false discovery cutoff for systemic errors. Second, the strain-specific effect could be due to cis-eQTL and tissue-specific effect could be due to trans-eQTL. Third, in comparison to the inbred mouse, the human is an outbred species and the complexity of haplotype phasing is much higher, which might lead to wrong read alignment (mapping bias). Since we cannot acquire a large sample size of SNPs calling data from the same population as the sampled genome, the only way to perform haplotype calling is to make inference via an established reference haploid genome from the same population. If the reference haploid genome distinctly differs from the sampled genome, then FDR inevitably rises. Recently, a Bayesian approach for analysis of ASE using a personal diploid genome as a reference sequence has been established and shows less biased alignments and more consistent ASE^56^. Fourth, small sample size and confounding underlying diseases (*e.g*. epilepsy, congenital deafness, or intellectual disability) may contribute to false detection. Fifth, in our study, we only had tissue from the prefrontal cortex of the offspring. However, other brain regions, such as the cerebellum, are also reported to be related to the anatomical and neuropathological causes of ASD^57^ and have different miRNA expression patterns^16^ in comparison with the prefrontal area.

Bipolar disorder was also present in the offspring with ASD. Because the age of onset for autism (2 to 3 years of age) is much earlier than that of bipolar disorder (25 years) and the offspring with ASD died at age 29 years, the impact of autism should be higher than that of bipolar disorder. In addition, many psychiatric disorders share genetic roots^58^. For example, there is an overlap between rare genetic variations linked to bipolar disorder and those implicated in autism^59^. Moreover, 83% of persons with ASD have been shown to have been diagnosed with at least one comorbid non-ASD developmental disorder^60^, therefore, it would be difficult to acquire cases of persons with ASD only. The results obtained here are more likely due to ASD rather than bipolar disorder.

Due to tissue availability, we sampled different regions of the prefrontal cortex (BA10 for the offspring without ASD and BA8 for the offspring with ASD) to conduct the analysis. The differential expression analysis may be confounded by the tissue specificity of gene expression in BA8 and BA10. To explore the impact of this potential confounding factor and verify the similarity of these two regions, we first compared the expression patterns of different regions of the central nervous system with principle component analysis (PCA) (Fig. S6a,b). PCA demonstrated similarities between BA8 and BA10 in comparison with the cerebellum and frontal cortex by PC2. It also showed a strong difference of BA8/10 from the amygdala, caudate nucleus, and spinal cord by PC1. Heatmap analysis again showed similarities between BA8 and BA10 in comparison to other regions of the central nervous system (Fig. S6c). To further confirm whether the differential gene expression in Figs 1b and 2a was due to brain region, we used heatmap and volcano analysis to examine those differentially expressed genes and observed that less than 7% of those genes were significantly affected (Fig. S6d to g and Supplementary Table 18). These findings show the two regions are similar, which supports our use of BA8 and BA10 for the comparison of gene expression.

In spite of these potential limitations and confounding factors, our results provide valuable clues for identifying biomarkers and biological signatures of ASD, and increase our understanding of potential underlying mechanisms that contribute to the pathogenesis of the disorder. Our findings not only advance our knowledge of allele-specific gene and miRNA expression in ASD, but also provide the first genomic map for allele-specific gene and miRNA expression in human PFC. These results could also provide clues to the evolutionary development of allele-specific expression. In addition, therapeutic targets and strategies for brain disorders such as ASD, as well as those involving ASE, could be determined. This will require development and validation of a plausible pipeline for ASE analysis in order to identify new genetic candidates for epigenetic mechanisms related to neuropathological characteristics of ASD, which could serve as targets for therapies of ASE-linked neurological disorders.

## Methods

### Subject material

We studied one family quartet comprised of parents and two offspring with and without ASD. In addition, one family trio, comprised of parents and one fetal offspring, was examined. Additional post-mortem samples included one fetal frontal cortex and one adult frontal cortex and cerebellum, shown in Supplementary Fig. 4. We assessed prefrontal cortex tissue from the dorsorostral pole of the frontal lobe corresponding to Brodmann’s area (BA) 10 for the offspring without ASD, and BA 8 for the offspring with ASD. Areas were based on tissue availability. Supplementary Fig. 1 and Supplementary Table 1 provides a more detailed pedigree of the family quartet showing epilepsy and deafness co-occurred in both the affected and unaffected offspring in addition to other familial health conditions. Moreover, we assessed prefrontal cortex tissue from the fetus and blood from the parents of the fetus. The detailed information of this family trio is shown in Supplementary Table 1. The detailed information of samples used for Supplementary Fig. 4 is shown in Supplementary Table 1. The institutional review boards (IRBs) of the participating institutions approved all experimental protocols. All experiments were carried out in accordance with the approved guidelines of the IRBs of the participating institutions. Written informed consent was obtained from both parents. Human quartet samples were obtained from the University of Utah Autism Research Program. The IRBs of the University of Utah, Icahn School of Medicine and National Taiwan University approved the analyses of samples and data in this study. Human trio samples and samples used for Supplementary Fig. 4 were obtained from National Taiwan University Hospital. The IRBs of the National Taiwan University approved the analyses of samples and data in this study. All tissue was fresh-frozen and stored at −80 degrees.

### RNA extraction and RNA sequencing (RNA-Seq)

Total RNA was extracted from postmortem PFC tissue using a NucleoSpin miRNA kit (Macherey-Nagel, 740971) according to the manufacturer’s instructions. The RNA was quantified with an ND-1000 spectrophotometer (Nanodrop Technology). The quality of RNA was based on the RNA integrity number (RIN) measured with a Bioanalyzer 2100 (Agilent Technology) and an RNA 6000 LabChip Kit (Agilent Technology). Ribosomal RNA was removed from the purified RNA with the Ribo-Zero Magnetic Gold Kit (Epicentre, MRZG126). Purified RNA was then amplified and prepared for sequencing with a SureSelect Strand-Specific RNA Library Prep Kit (Agilent Technologies, G9691A). Libraries were sequenced using sequencing-by-synthesis technology (TruSeq SBS Kit v3-HS, Illumina, FC-401-3001) on an Illumina HiSeq. 2000 (100 base pairs paired-end reads) at the Welgene Biotech Company, which generated 6 Gb reads of data per sample. The detailed information of RNA-Seq is shown in Supplementary Table 1.

### Detection of variants

DNA from the parents and offspring was extracted and sequenced according to standard whole-genome sequencing protocol. The sequencing reads were trimmed with Trimmomatic to obtain the qualified reads. The reads were then aligned to the human reference genome GRCh38 using BWA and processed with SAMtools. Picard (http://broadinstitute.github.io/picard/) was implemented to mark the duplicate reads and exclude them from downstream analyses. The read alignments were further refined with GATK for local realignment of reads around known insertions and deletions (indels) and recalibration of base quality. GATK was also applied to call single-nucleotide variants (SNVs) and short indels. Data from SNVs were consequently used to construct the haplotype scaffolds as described below. The RNA-Seq and DNA-Seq data discussed in this publication have been deposited in NCBI’s Gene Expression Omnibus and are accessible through GEO Series accession number GSE98581.

### Allele-specific expression analysis

The SNV data from the parents and offspring were processed with VCFtools. SHAPEIT was then used to phase the SNV data with the family pedigree for building phased haplotype scaffolds. To improve phasing accuracy, the information of recombination rates between SNPs was provided via a genetic map retrieved from The 1000 Genomes Project Phase 3. The reference panel of phased haplotypes belonging to Utah residents with Northern and Western European ancestry (CEU) from The 1000 Genomes Project Phase 3 was also applied to align SNPs between the dataset and the panel for assisting in reliable phasing. The information of phased haplotypes was subsequently analyzed with in-house scripts to create haploid genomic sequences for the parents and offspring based on the human reference genome GRCh38. For RNA-Seq analysis, the sequences generated were filtered to obtain qualified reads. ConDeTri was implemented to trim or remove the reads according to the quality score. Qualified reads after filtering low-quality data were analyzed using TopHat/Cufflinks for gene expression estimation. The gene expression level was calculated as FPKM (Fragments Per Kilobase of transcript per Million mapped reads). A comprehensive analysis of the tissue using mammalian transcriptome data sets suggests that a lower cutoff of FPKM = 0.3 is often justifiable therefore we applied this cutoff for all analysis of mammalian transcriptomes. For differential expression analysis, CummeRbund was employed to perform statistical analyses of gene expression profiles. For allele-specific expression analysis, MMSEQ was then implemented to estimate allelic imbalance and deconvolve the alignment of reads to diploid transcripts derived from diploid genomic sequences and Ensembl gene annotation 74 following the mapping of RNA-Seq reads with Bowtie. SNPs for confirming imprinted miRNAs are within primary miRNA sequence. We defined the sequence for primary miRNAs as the genomic locus from 500 bp upstream to 500 bp downstream of the mature miRNA sequence. The FPKM value is much less than from regular read analysis because only the read with SNPs can be used for allele-specific expression analysis.

### Reverse transcription quantitative PCR (RT-qPCR)

Total RNA was extracted from the postmortem PFC of the offspring using a NucleoSpin® miRNA kit (Macherey-Nagel, 740971). Total RNA (10 ng) was converted to cDNA and amplified by One Step SYBR® PrimeScriptTMRT-PCR Kit II (Takara, PR086A). Quantitative real-time PCR was performed with a StepOnePlus Real Time PCR System (Applied Biosystems). Ct values were generated using StepOne Software version 2.2.2. The expression level of each gene was normalized to *B2M*. All primer sequences of candidate genes were designed by Primer3 software (http://bioinfo.ut.ee/primer3-0.4.0/) and are shown in Supplementary Table 19.

### miRNA quantification

We extracted miRNA using a NucleoSpin® miRNA kit (Macherey-Nagel, 740971). Because the length of miRNA is too short to perform normal qRT-PCR, the miRNA was lengthened with a Poly(A) tail (Poly(A) Tailing Kit; Ambion, AM1350). The poly(A) tailed miRNA was reverse transcribed into cDNA with a poly(T) anchor adaptor. The miRNA was amplified and quantitated by qPCR using a specific miRNA forward primer and a universal adaptor primer. Information of primer sequence is shown in Supplementary Table 20.

Similarly, the qPCR product was too small for Sanger sequencing. Therefore, to determine the sequence, the amplified PCR product was inserted into a plasmid vector, and the vector was transformed into bacteria with the TOPO TA Cloning Kit (Invitrogen, 450071), cloned, and cultured according to the manufacturer’s directions. Plasmid DNA containing the inserted qPCR product was extracted with the Presto Mini Plasmid kit (Geneaid, PHD100). Sanger sequencing of the purified plasmid used M13-tailed primers, which can yield sequences up to approximately 200 bp.

### Graphic representation and statistical analysis

Heatmaps were generated with Pretty Heatmaps software (pheatmap package in R 3.3.2). The hierarchical clustering of heatmaps and the supplementary tables were measured in Euclidean distance. Micro-RNA target prediction was performed via DIANA microT-CDS web-based program. Pedegree was generated with Genial Pedigree Draw (www.pedigreedraw.com). Gene ontology (GO) analysis were performed via web-based Gorilla program and miEAA program for gene set enrichment analysis (GSEA) adapted for miRNA. Statistical analysis and graphic illustrations were performed using R 3.3.2 and Sigmaplot 13.0. All values with technical triplicates are expressed as the mean ± standard error of mean (s.e.m.).

### Data availability statement

All data are available in this manuscript.

## Electronic supplementary material

Supplementary information

Table S1

Table S2

Table S3

Table S4

Table S5

Table S6

Table S7

Table S8

Table S9

Table S10

Table S11

Table S12

Table S13

Table S14

Table S15

Table S16

Table S17

Table S18

Table S19

Table S20
