# Supplementary material for: Allele-specific expression in a family quartet with autism reveals mono-to-biallelic switch and novel transcriptional processes of autism susceptibility genes
Source: Sci Rep. 2018 Mar 9;8:4277. doi: 10.1038/s41598-018-22753-4 (PMC5844893; doi:10.1038/s41598-018-22753-4)
Supplement: Supplementary file 1 — Supplementary information [file 41598_2018_22753_MOESM1_ESM.pdf]

Allele-specific Expression in a Family Quartet with Autism Reveals Mono-to-biallelic Switch and Novel Transcriptional Processes of Autism Susceptibility Genes

Chun-Yen Lin[1,2], Kai-Wei Chang[1], Chia-Yi Lin[1], Jia-Ying Wu[1], Hilary Coon[3], Pei-Hsin Huang [4], Hong-Nerng Ho[5,6], Schahram Akbarian[7], Susan Shur-Fen Gau[1,8], and Hsien-Sung Huang [1,9,#]

SUPPLEMENTARY METHOD, FIGURE AND TABLE LEGENDS AND REFERENCES

Analysis of different regions of normal human postmortem brains and spinal cords. Samples from normal human subjects are as follows: BA8 of prefrontal cortex is from GSE102556; BA10 of prefrontal cortex is from GSE17612; cerebellum and frontal cortex are from GSE36192, 20 random samples of each, out of > 400 samples each); amygdala, caudate nucleus and spinal cord are from GSE2361. Microarray samples were normalized by subtracting background signals. For the RNA-seq samples, the minimum expression was arbitrarily set to 0.001 FPKM. Genes listed in all platforms were used for analysis. We used centered $\log_2$ signal to evaluate data distribution for cross-platform comparativity. The samples were then quantile normalized. Principal component analysis

(PCA), heatmap plots and volcano plot analysis via RStudio[1] were used to evaluate the similarity between BA8 and BA10 regions.

Comparison of our altered genes and miRNAs between our study cohort and another ASD cohort. The other ASD cohort was obtained from Dr. Weinberger's group[2]. We used GRCh38 reference genome and Ensembl 90 for transcriptomic analysis. We used the STAR 2.5.1 program for mapping. The Cuffdiff program was used for expression analysis.

Supplementary Figure 1. The detailed pedigree of the family quartet with ASD. Square shape indicates male and circle shape indicates female. Diamond shape indicates unknown gender. The quartet samples for this study are highlighted with a red rectangle.

Supplementary Figure 2. (**a**) Quality of DNA from each family member was determined using an Agilent 2100 Bioanalyzer. The ratio of Qubit to NanoDrop concentrations was larger than 0.67 for all four samples. Concentration of DNA was > 5 μg for all four samples. Y-axis indicates the sample intensity. (**b**) RNA profiles for the two offspring were generated on an Agilent 2100 Bioanalyzer to determine RNA integrity number (RIN). The RIN was 5.6 for the non-ASD offspring and 5.1 for the offspring with ASD, which was greater than the minimal RIN (3.95) for good quality human postmortem brain RNA[3,4]. The RNA concentration used was > 5 μg for the two offspring. The Y-axis indicates the sample intensity.

Supplementary Figure 3. Comparison of expression of altered genes and miRNAs in our study cohort and another ASD cohort. (**a**) Altered genes are from Fig. 1b. (**b**) Altered miRNAs are from Fig. 1e. (**c**) Altered genes are from Fig. 2a. ASD and Non-ASD

are samples from our cohort. C_ASD and C-Non ASD are samples from Dr. Weinberger's group.

Supplementary Figure 4. Confirmation of allele-specific expression in postmortem brains. Sanger sequencing of (a) *LRP2BP* in fetal cerebral cortex (C.C.) and adult cerebellum (C.B.) and frontal cortex (F.C.) (b) *ATP10A*, *DLGAP2*, *HTR2A* and *DUSP22* in fetal cerebral cortex (C.C.).

Supplementary Figure 5. Confirmation of known human imprinted genes in the postmortem PFC of the offspring without ASD. PEG = paternally expressed gene. MEG = maternally expressed gene. "Consistent" indicates the imprinting status was the same as that of known imprinted genes. "Inconsistent" indicates the imprinting status was not the same as that of known imprinted genes or could not be interpreted due to lack of SNPs.

Supplementary Figure 6. Transcriptomic comparison between human BA8 and BA10. (**a**) Data distribution prior to cross-platform normalization. (**b**) PCA analysis of different regions of the central nervous system. (**c**) Heatmap of all genes in the tested samples. (**d**) (Left) heatmap of all up-regulated genes (n=1,069) shown in fig. 1b. (Right) heatmap of all up-regulated ASD-susceptibility genes (n=89) shown in fig. 2a. (**e**) Volcano plot analysis of up-regulated normal and ASD-susceptibility genes. (**f**) (Left) heatmap of all down-regulated genes (n=2,731) shown in fig. 1b. (Right) heatmap of all down-regulated ASD-susceptibility genes (n=185) shown in fig. 2a. (**g**) Volcano plot analysis of down-regulated normal and ASD-susceptibility genes. FC: fold change; FDR: false discovery rate. Genes which were not expressed (mean expression < 0.1 normalized signal or FPKM in both samples) were discarded.

Supplementary Table 1. Summary of the family quartet with ASD, the family trio and tissues for supplementary figure 4

Supplementary Table 2. Gene information for heatmap clustering

Supplementary Table 3. Summary of GO analysis of altered genes

Supplementary Table 4. Gene information for stringent criteria

Supplementary Table 5. Gene information of the comparison between our study ASD cohort and raw data of an ASD cohort obtained from Dr. Weinberg's group

Supplementary Table 6. Gene information of the comparison between our study ASD cohort and raw data of an ASD cohort obtained from Dr. Geschwind's group

Supplementary Table 7. miRNA information for heatmap clustering

Supplementary Table 8. Summary of GO analysis of altered miRNAs

Supplementary Table 9. List of targeted genes by altered miRNAs

Supplementary Table 10. List of autism susceptibility genes from SFARI

Supplementary Table 11. Gene information for heatmap clustering

Supplementary Table 12. List of autism susceptibility miRNAs and their target genes

Supplementary Table 13. Ratios of ASE of all genes

Supplementary Table 14. Gene information of heatmap clustering

Supplementary Table 15. List of imprinted genes from Geneimprint website

Supplementary Table 16. miRNA information for heatmap clustering

Supplementary Table 17. List of confirmed imprinted genes in postmortem PFC of the offspring without ASD

Supplementary Table 18. Gene information for transcriptomic comparison between human BA8 and BA10 (supplementary figure 6)

Supplementary Table 19. Primer information for genes

Supplementary Table 20. Primer information for miRNAs

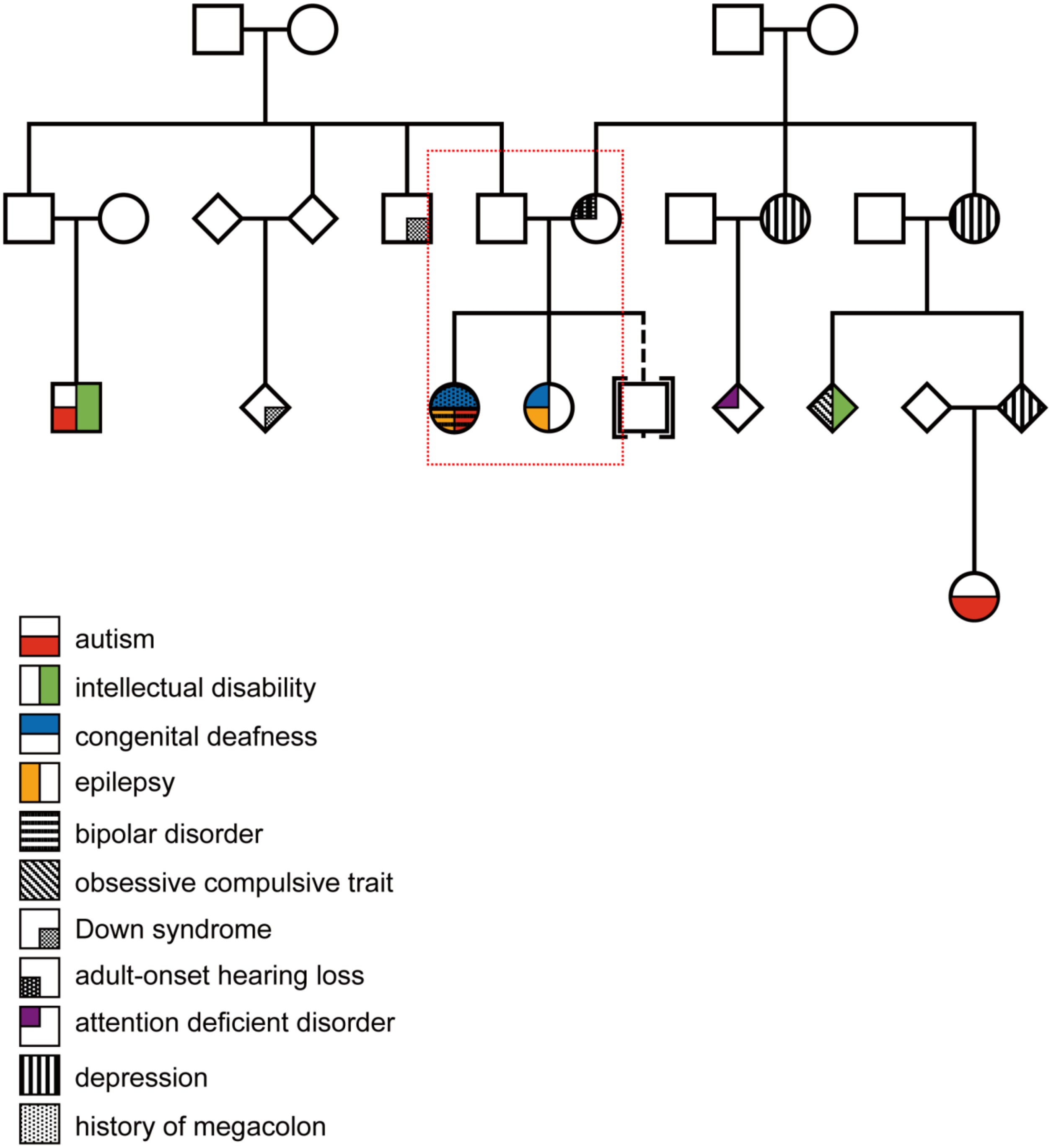

autism
intellectual disability
congenital deafness
epilepsy
bipolar disorder
obsessive compulsive trait
Down syndrome
adult-onset hearing loss
attention deficient disorder
depression
history of megacolon

Figure S1

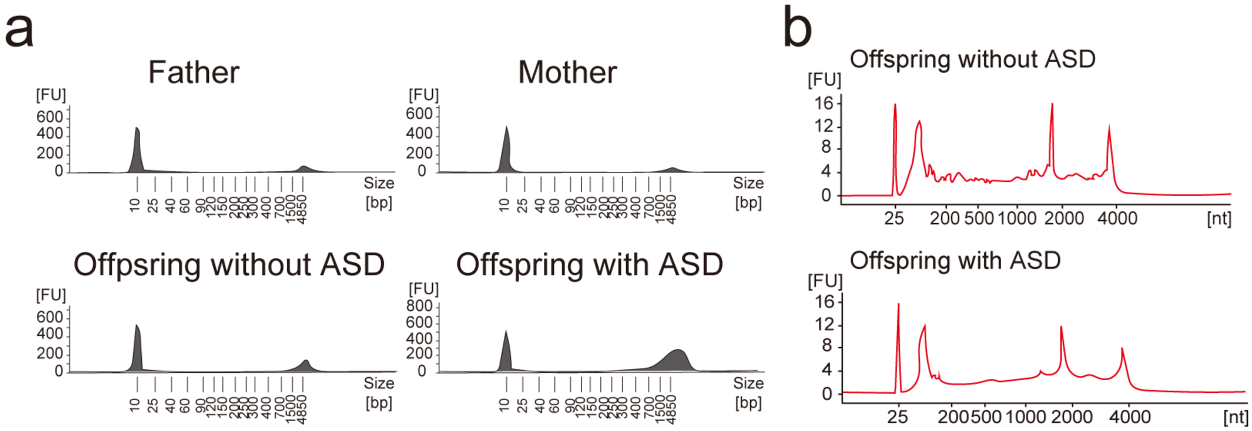
a
Father
Mother
Offpsring without ASD
Offspring with ASD
[FU]
Size
[bp]
10 25 40 60 90 120 150 200 250 300 400 700 1500 4850
b
Offspring without ASD
Offspring with ASD
[FU]
0 4 8 12 16
25 200 500 1000 2000 4000 [nt]

Figure S2

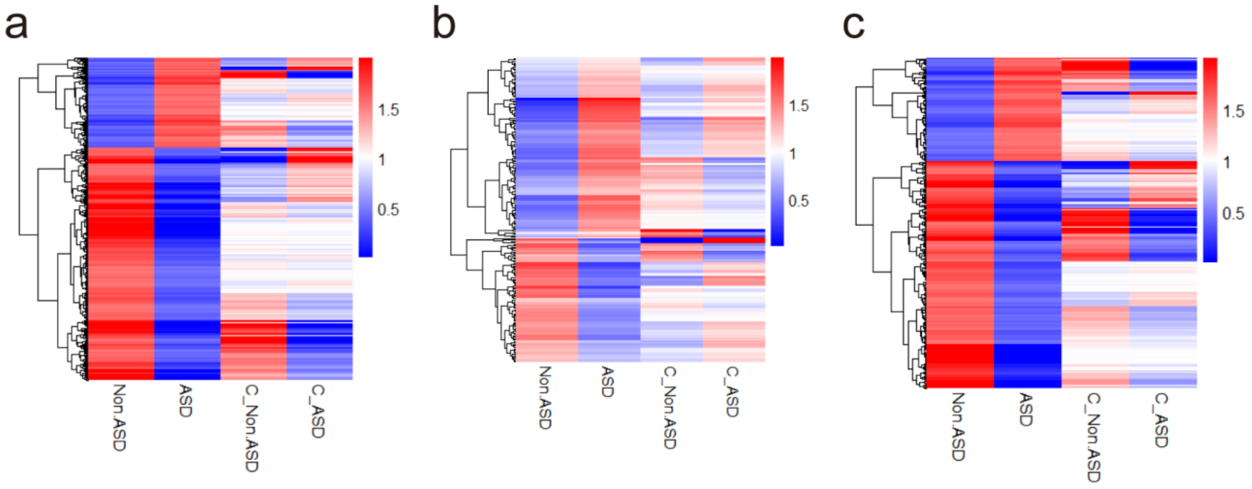

Figure S3

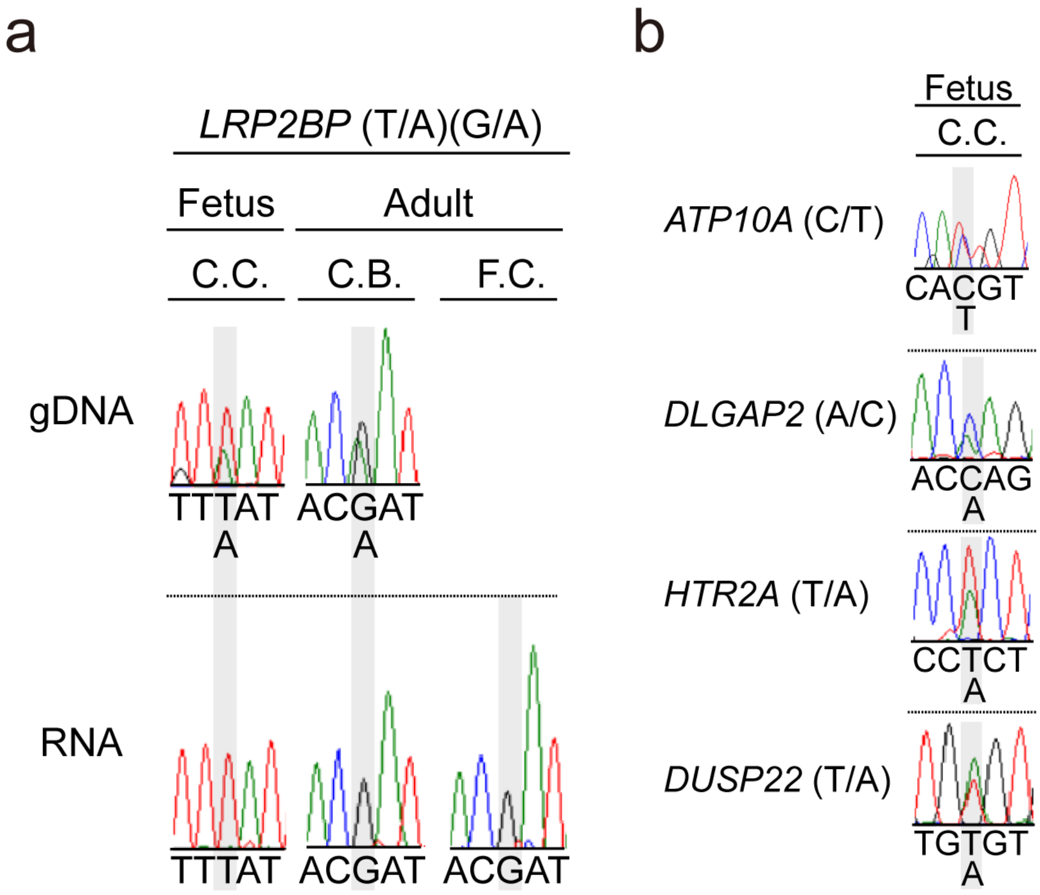
a
LRP2BP (T/A)(G/A)
Fetus
Adult
C.C.
C.B.
F.C.
gDNA
TTTAT
A
ACGAT
A
RNA
TTTAT
ACGAT
ACGAT
b
Fetus
C.C.
ATP10A (C/T)
CACGT
T
DLGAP2 (A/C)
ACCAG
A
HTR2A (T/A)
CCTCT
A
DUSP22 (T/A)
TGTGT
A

Figure S4

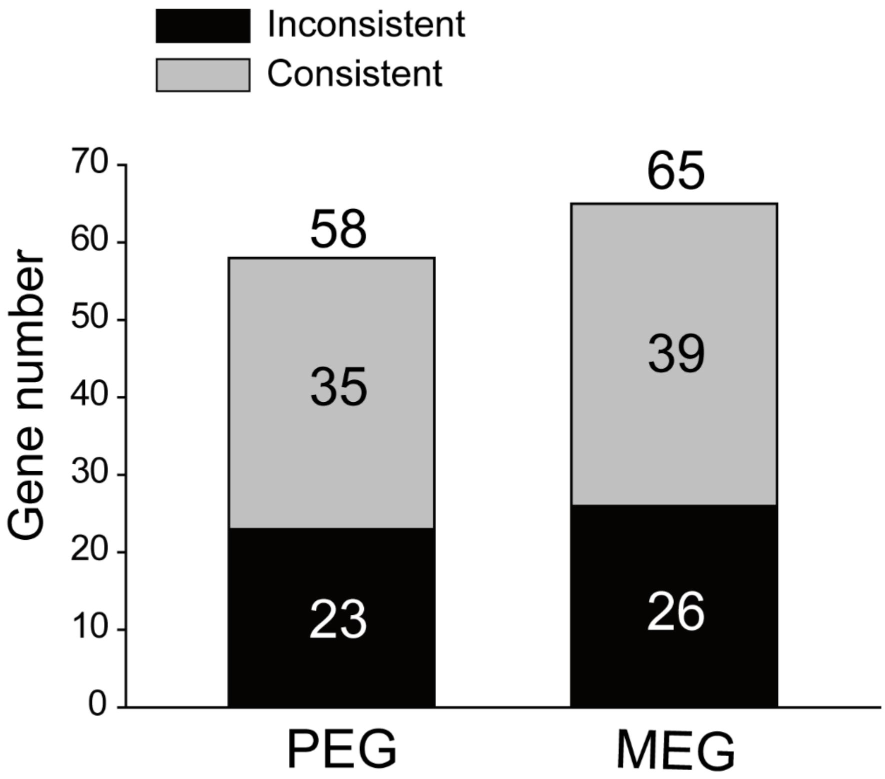
Inconsistent
Consistent
Gene number
70
60
50
40
30
20
10
0
58
65
35
39
23
26
PEG
MEG

Figure S5

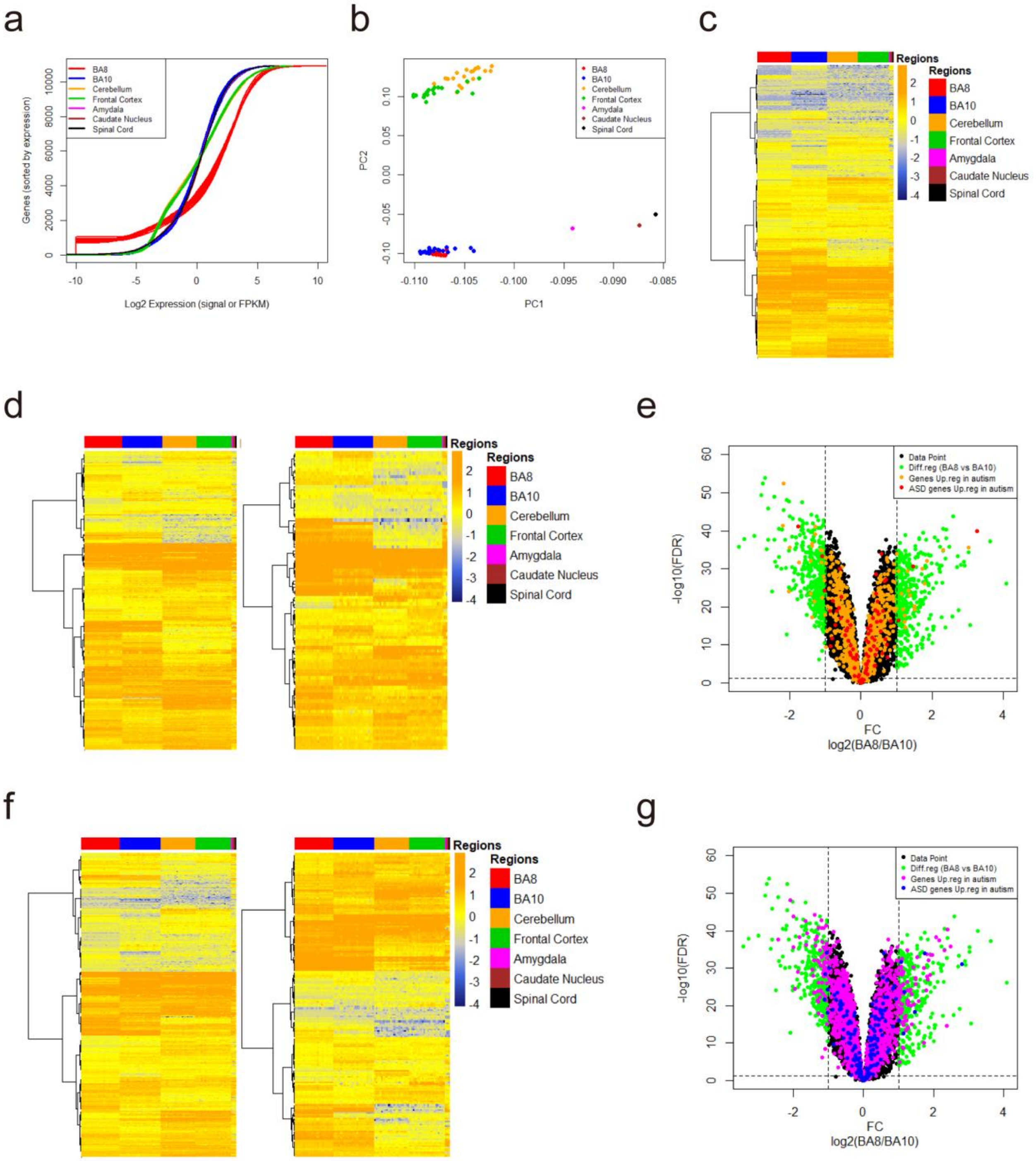
a
b
c
d
e
f
g
BA8
BA10
Cerebellum
Frontal Cortex
Amygdala
Caudate Nucleus
Spinal Cord
Genes (sorted by expression)
Log2 Expression (signal or FPKM)
PC1
PC2
Regions
Data Point
Diff.reg (BA8 vs BA10)
Genes Up.reg in autism
ASD genes Up.reg in autism
-log10(FDR)
FC
log2(BA8/BA10)

Figure S6

References:

1 R: A language and environment for statistical computing. (R Foundation for Statistical Computing, Vienna, Austria., 2016).

2 Wright, C. *et al.* Altered expression of histamine signaling genes in autism spectrum disorder. *Translational psychiatry* **7**, e1126, doi:10.1038/tp.2017.87 (2017).

3 Weis, S. *et al.* Quality control for microarray analysis of human brain samples: The impact of postmortem factors, RNA characteristics, and histopathology. *Journal of neuroscience methods* **165**, 198-209, doi:10.1016/j.jneumeth.2007.06.001 (2007).

4 Koppelkamm, A., Vennemann, B., Lutz-Bonengel, S., Fracasso, T. & Vennemann, M. RNA integrity in post-mortem samples: influencing parameters and implications on RT-qPCR assays. *Int J Legal Med* **125**, 573-580, doi:10.1007/s00414-011-0578-1 (2011).
